# Preoperative prediction of early recurrence of HBV-related hepatocellular carcinoma (≤5 cm) by visceral adipose tissue index

**DOI:** 10.3389/fsurg.2022.985168

**Published:** 2023-01-06

**Authors:** Zong-qian Wu, Jie Cheng, Xi-xi Xiao, Hua-rong Zhang, Jian Wang, Juan Peng, Chen Liu, Ping Cai, Xiao-ming Li

**Affiliations:** ^1^ Department of Radiology, Southwest Hospital, Third Military Medical University (Army Medical University), Chongqing, China; ^2^ Department of Oncology and Southwest Cancer Center, Southwest Hospital, Third Military Medical University (Army Medical University), Chongqing, China; ^3^ Institute of Pathology and Southwest Cancer Center, Southwest Hospital, Third Military Medical University (Army Medical University), Chongqing, China; ^4^ Department of Radiology, The First Affiliated Hospital, Chongqing Medical University, Chongqing, China

**Keywords:** visceral adipose tissue, HBV, HCC, early recurrence, abdominal adipose tissue, computed tomography

## Abstract

**Background:**

This study aimed to investigate whether visceral adipose tissue index (VATI) is a significant risk factor for the early recurrence (ER) of HBV-related hepatocellular carcinoma (HCC) (≤5 cm) after hepatectomy.

**Methods:**

The recruited cohort patients who were positive for hepatitis B virus, presented with surgically confirmed HCC (≤5 cm) from Army Medical University (internal training cohort: *n* = 192) and Chongqing Medical University (external validation group: *n* = 46). We measured VATI, subcutaneous adipose tissue index (SATI) *via* computed tomography (CT). ER was defined as recurrence within 2 years after hepatectomy. The impact of parameters on outcome after hepatectomy for HCC was analyzed.

**Results:**

Univariate analysis showed that alpha-fetoprotein levels (*p* = 0.044), body mass index (BMI) (*p* < 0.001), SATI (*p* < 0.001), and VATI (*p* < 0.001) were significantly different between ER and non-ER groups in internal training cohort. Multivariate analysis identified VATI as an independent risk factor for ER (odds ratio = 1.07, 95% confidence interval: 1.047–1.094, *p* < 0.001), with a AUC of 0.802, based on the cut-off value of VATI, which was divided into high risk (≥37.45 cm^2^/m^2^) and low risk (<37.45 cm^2^/m^2^) groups. The prognosis of low risk group was significantly higher than that of high risk group (*p *< 0.001). The AUC value of VATI in external validation group was 0.854.

**Conclusion:**

VATI was an independent risk factor for the ER, and higher VATI was closely related to poor outcomes after hepatectomy for HBV-related HCC (≤5 cm).

## Introduction

Hepatocellular carcinoma (HCC) is the most common primary malignancy of the liver, accounting for approximately 80%–90% of all liver malignant tumors (1). It is also the fifth most common cancer and a leading cause of cancer-related deaths worldwide, with approximately 50% of cases occurring in China (2–4). In eastern Asia and Africa, hepatitis B virus (HBV) related-HCC is the predominant etiological factor. As suggested by clinical practice guidelines of American Association for the Study of Liver Diseases (AASLD) (5) and National Comprehensive Cancer Network (NCCN) (6), radiofrequency ablation and radical resection remain primary treatments for patients with early HCC. However, about 30% of patients are still free of disease recurrence (7).

The recurrence of HCC is classified as early recurrence (ER) (within 2 years) and non-ER (after 2 years) based on the time of recurrence after hepatectomy (2, 8). It has been reported that approximately 80% of early HCC recurrence occurs in the liver (8, 9). Furthermore, there are many risk factors for postoperative HCC recurrence including postoperative residual tumor tissuett, microvascular invasion, severe cirrhosis, portal hypertension, DNA replication, *α*-fetoprotein (AFP), and protein-induced by vitamin K absence-II (2, 10–13).

Lately, obesity and its associated metabolic abnormalities have been reported in previous studies as risk factors for poor prognosis in certain malignancies, including colon, endometrial, renal, and pancreatic cancers (14–16). Abdominal adipose tissue, including visceral adipose tissue (VAT) and subcutaneous adipose tissue (SAT), which are significantly related to obesity and metabolic syndrome. Therefore, we hypothesized that abdominal adipose tissue is an important risk factor for ER after HCC resection. CT imaging was used to quantify abdominal adipose tissue in a rapid and precise technique.

As there have been no report regarding whether abdominal adipose tissue is a risk factor for ER after HBV-related HCC resection, the main objective of this study was to investigate the correlation between abdominal adipose tissue and ER of HBV-related HCC after hepatectomy.

## Materials and methods

### Patient population

This study has been approved by the Ethics Committee of the First Affiliated Hospital (Army Medical University) (KY2021088) and the requirement for informed consent was waived. The study protocol conforms to the ethical guidelines of the 1975 Declaration of Helsinki as reflected in *a priori* approval by the institution's human research committee. We collected the patients with HBV-related HCC in Army Medical University (internal training cohort) from January 2018 to October 2020, and Chongqing Medical University (external validation cohort) from January 2018 to January 2019.

The inclusion criteria were as follows: (1) pathological confirmation of HCC after surgical resection; (2) availability of complete clinical data; (3) CT performed within two weeks before surgery; (4) only a single tumor present (diameter ≤ 5 cm), excluding tumors with surrounding satellite lesions; (5) no intra or external metastasis and no visible vascular invasion; and (6) no preoperative history of other liver treatments, including radiofrequency ablation, liver resection, transcatheter arterial chemoembolization, or liver transplantation.

The exclusion criteria were as follows: (1) incomplete clinical data; (2) tumor diameter (>5 cm); (3) prior hepatic treatments before hepatectomy including radiofrequency therapy, transcatheter arterial chemoembolization, resection, or liver transplantation; (4) multiple tumors (*n* ≥ 2); (5) interval between the surgery date and CT examination was greater than two weeks; (6) poor image quality.

### Image acquisition

The CT images of patients were collected using a dual-source CT scanner (SOMATOM Definition Flash, Siemens, Germany) and spiral CT scanner (Light Speed, GE, American). The scan range extended from the top of the diaphragm to the 4th lumbar vertebra (L4). Non-contrast scan followed by contrast-enhanced CT scans were acquired in the axial plane. Scan parameter settings were as follows: tube voltage of 120 kV, with automatic tube current, a layer thickness of 5 mm and a 1.5 mm reconstruction interval.

### Image analysis

**Figure 1:**
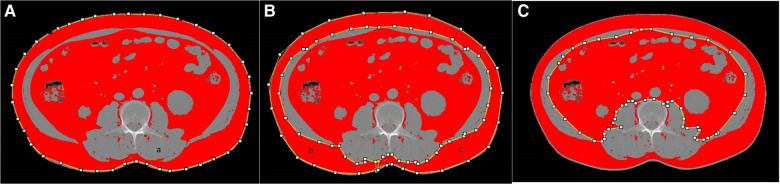
Example images of abdominal adipose tissue as measured by using imageJ software. total adipose (**A**), subcutaneous adipose (**B**), visceral adipose (**C**).

Abdominal adipose tissue were semi-automatically measured on 1.5 mm thick non-enhanced CT images using ImageJ software (https://imagej.net/software/fiji/-downloads). SAT and VAT areas were measured in adjusted-tissue Hounsfield units (HU) as follows: −190 to −30 HU. One radiologist with 10 years of experience in abdominal imaging, blinded to patients' information, evaluated cross-sectional areas of abdominal adipose tissue. A single unenhanced axial slice (layer thickness of 1.5 mm) at the L3 vertebral center was selected independently, the abdominal adipose tissue was delineated three times using ImageJ software and the average values were recorded (Figure 1). Then we used the abdominal adipose tissue divided by height (cm^2^/m^2^) to obtain the VATI and SATI as reported in the literature (17).

Patients with HBV-related HCC underwent routine imaging (ultrasound, CT, and/or MRI) and AFP testing at 3, 6, 12, and 24 months after hepatectomy. ER was established on typical findings on contrast-enhanced multiphase imaging [hyperenhancement in the arterial phase and washout in the portal venous or delayed phases (18)] within two years after surgery, including intrahepatic recurrence or distant metastases (19), otherwise was non-ER. The observational period for recurrence analysis was defined as the interval between surgery and recurrence or the date of the last follow-up visit before May 30, 2022.

### Statistical analyses

Statistical analyses were performed using SPSS 22.0 software (Armonk, NY: IBM Corp). Variables with normal distributions are expressed as (¯x ± s), and pairwise comparisons were performed using the Student's *t* test. Nonnormally distributed continuous variables were analyzed using the Mann-Whitney test, while categorical variables were analyzed using the *χ*^2^-test. Univariate logistic regression was used to analyze the prognostic factors, variables with *p* < 0.05 were included in bivariate logistic regression analysis. Receiver operating characteristic curves were also plotted to establish VATI cut-off values associated with ER, and divided into low risk and high risk groups. RFS in the low risk and high risk was analyzed using Kaplan-Meier curves and compared using a log-rank test. For all analyses, differences with *p *< 0.05 were considered statistically significant.

## Results

### Patient characteristics

**Figure 2:**
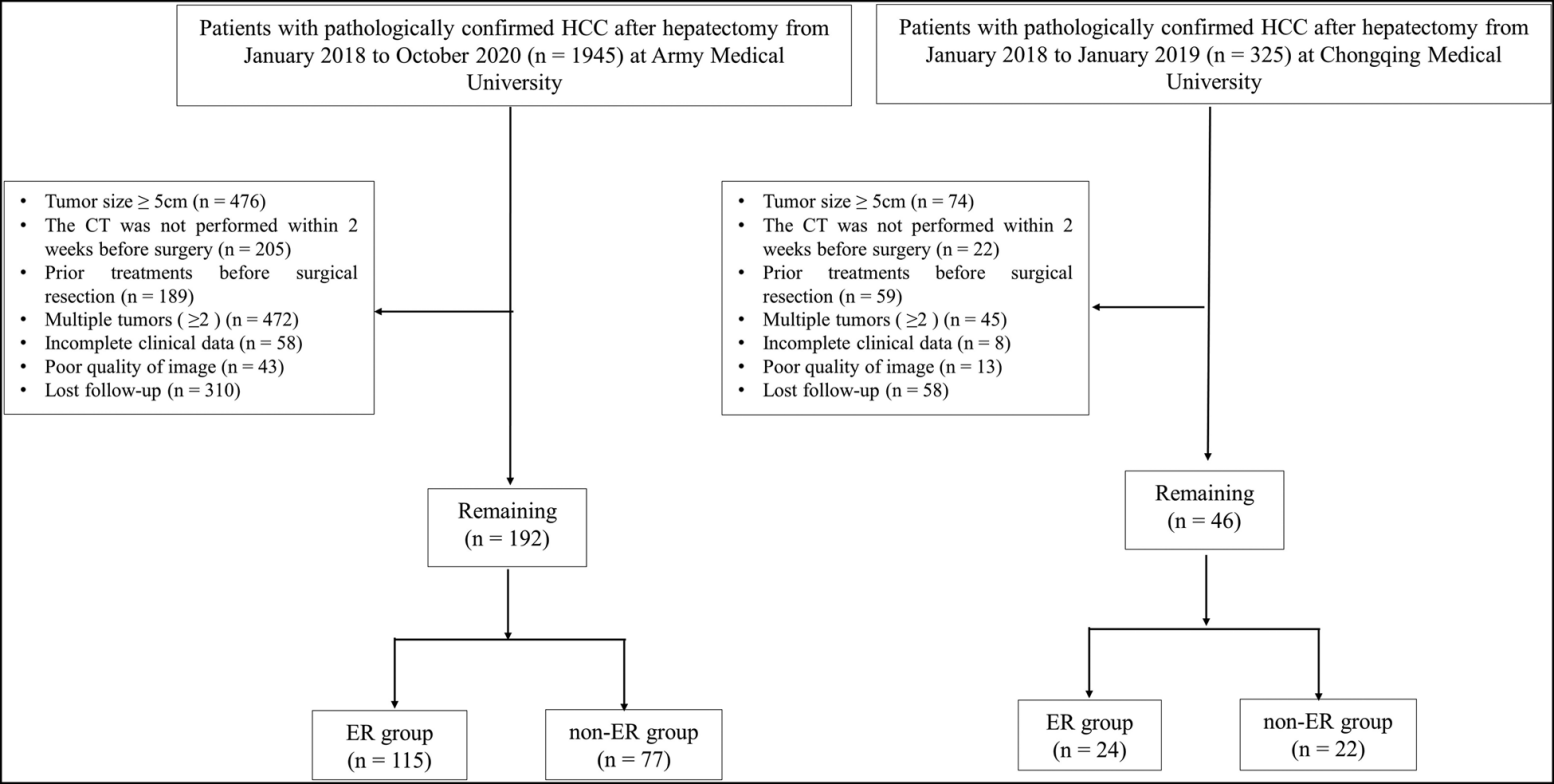
Flowchart of retrospective patient selection of HBV-related HCC in two cohort.

A total of 238 patients were included in the present study, including Army Medical University (internal training cohort) and Chongqing Medical University (external validation cohort) (Figure 2). The internal training cohort comprised 192 patients with a male majority population (85.9%), and the external validation cohort comprised 46 patients with a male majority population (91.3%). Body mass index (BMI), SATI and VATI were similar in the two cohorts (Table 1).

**Table 1:** Baseline characteristics of the patients.

Variable	Army Medical University (*n *= 192)	Chongqing Medical University (*n *= 46)	*t/z/χ^2^*	*p*
Sex (*n*)				
Male	165 (85.9%)	42 (91.3%)	0.944	0.331
Female	27 (14.1%)	4 (8.7%)		
BMI (kg/m^2^)	23.2 ± 2.8	23.2 ± 3.2	0.054	0.957
Age, years	50.6 (44.0, 56.8)	55.4 (45.0, 65.3)	−2.766	0.006
Drinking history (*n*)				
Present	56 (29.2%)	23 (50.0%)	7.263	0.007
Absent	136 (70.8%)	23 (50.0%)		
Smoking history (*n*)				
Present	87 (45.3%)	29 (63.0%)	4.670	0.031
Absent	105 (54.7%)	17 (37.0%)		
DM (*n*)				
Present	22 (11.5%)	7 (15.2%)	0.490	0.484
Absent	170 (88.5%)	39 (84.8%)		
Liver cirrhosis (*n*)				
Present	136 (70.8%)	33 (71.7%)	0.015	0.903
Absent	56 (29.2%)	13 (28.3%)		
Child-pugh score (*n*)				
A	186 (96.9%)	46 (100%)	1.475	0.225
B	6 (3.1%)	0 (0.0%)		
Tumor size (cm)	3.1 (2.2, 4.0)	3.2 (2.1, 4.3)	−0.649	0.516
Albumin (g/L)	43.4 (41.2, 45.9)	43.3 (38.0, 46.2)	−1.027	0.305
Platelet count (10^9^/dl)	140.9 (97.8, 173.0)	142.9 (102, 170.5)	−0.358	0.721
AFP (ng/ ml)	614.9 (5.4, 193.9)	470.2 (5.3, 376.6)	−0.358	0.738
TBIL (μmol/L)	20.7 (12.7, 20.7)	14.8 (11.3, 17.3)	−2.216	0.027
BCLC stage (n)				
0	39 (20.3%)	21 (45.7%)	12.638	<0.001
A	153 (79.7%)	25 (54.3%)		
Antiviral therapy (*n*)				
Present	70 (36.5%)	8 (17.4%)	6.123	0.013
Absent	122 (63.5%)	38 (82.6%)		
Surgical approach (*n*)				
Laparotomy	70 (36.5%)	29 (63.0%)	10.796	0.001
Laparoscopic	122 (63.5%)	17 (37.0%)		
Type of resection (*n*)				
AR	104 (54.2%)	21 (45.7%)	1.079	0.299
Non-AR	88 (45.8%)	25 (54.3%)		
SATI (cm^2^/m^2^)	43.5 (28.5, 53.7)	39.8 (25.4, 50.7)	−0.997	0.319
VATI (cm^2^/m^2^)	43.7 (25.0, 58.6)	53.4 (25.5, 71.6)	−1.783	0.075

AFP, *α*-Fetoprotein; TBIL, total bilirubin; HBV, hepatitis B virus; DM, diabetes mellitus; BMI, body mass index; AR, anatomical resection; non-AR, non-anatomical resection; VATI, visceral adipose tissue index; SATI, subcutaneous adipose tissue index; BCLC, Barcelona Clinic Liver Cancer.

### Association between parameters and early recurrence (ER) in internal training cohort

In internal training cohort, patients were divided into ER group (*n* = 115) and non-ER group (*n* = 77). Univariate analysis demonstrated clinical factors, except for AFP (*p *= 0.044) and (BMI) (*p *< 0.001), no variables showed significant differences between the ER and non-ER groups (*p* > 0.05).

**Figure 3:**
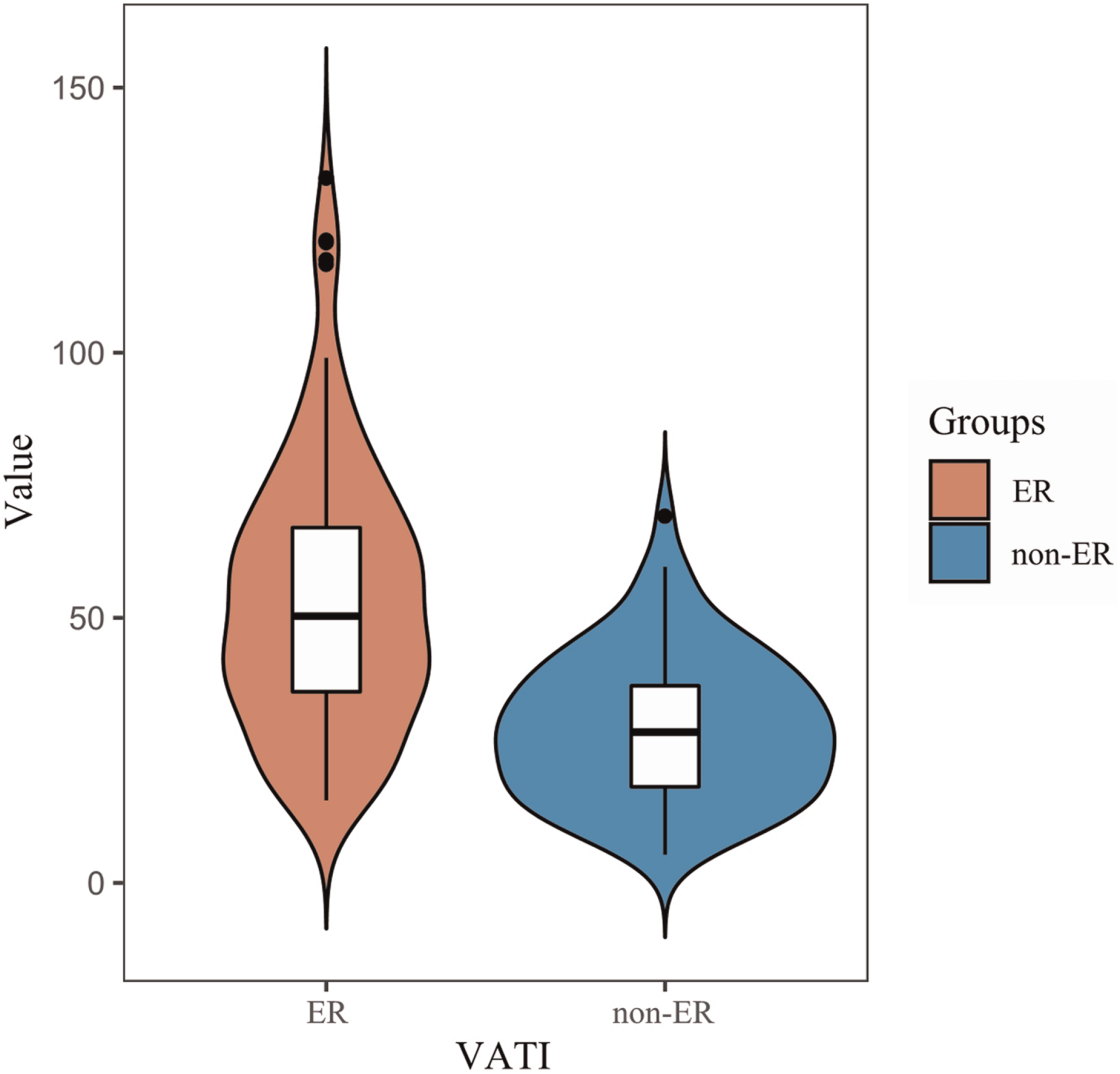
Comparison of the VATI between the ER and non-ER groups. An increase in visceral adipose tissue index (VATI) value was associated with an increased risk of early recurrence.

Univariate analysis showed among VATI, SATI with ER group was higher than that of non-ER group [53.5 ± 2.7 cm^2^/m^2^ vs. 29.4 ± 13.7 cm^2^/m^2^, 45.7 cm^2^/m^2^ (33.6, 58.5) vs. 32.5 cm^2^/m^2^; *p *< 0.001]. Multivariate analysis showed that VATI was the only an independent risk factor (odds ratio = 1.07, 95% confidence interval: 1.047–1.094; *p *< 0.001) for ER (Table 2) (Figure 3).

**Table 2:** Risk factors for early recurrence after hepatectomy.

			Univariate		Multivariate
Variable	Recurrence (*n *= 115)	No-recurrence (*n *= 77)	*t/z/χ^2^*	*p*	*p*
BMI (kg/m^2^)	24.0 ± 2.7	22.0 ± 2.5	5.41	<0.001	0.485
Sex(*n*)					
Male	101 (87.8%)	64 (83.1%)	0.849	0.358	
Female	14 (12.2%)	13 (12.9%)			
Age, years	50.0 (44.0, 57.3)	50 (43.2, 54.0)	−1.35	0.177	
Drinking history(*n*)					
Present	37 (32.2%)	19 (24.7%)	1.255	0.263	
Absent	78 (67.8%)	58 (75.3%)			
Smoking history(*n*)					
Present	56 (48.7%)	31 (40.2%)	1.324	0.25	
Absent	59 (51.3%)	46 (59.8%)			
DM (*n*)					
Present	17 (14.7%)	5 (6.4%)	3.123	0.077	
Absent	98 (85.2%)	72 (95.3%)			
Liver cirrhosis (*n*)					
Present	80 (69.6%)	55 (71.4%)	0.077	0.782	
Absent	35 (30.4%)	22 (28.6%)			
AFP (ng/ ml)	36.1 (7.6, 193.8)	8.2 (4.0, 265.8)	−2.014	0.044	0.719
Albumin(g/L)	43.5 (40.9, 45.9)	43.8 (41.4, 45.9)	−0.729	0.466	
Tumor size (cm)	3.2 (2.2, 4.0)	2.8 (2.0, 3.9)	−1.017	0.309	
TBIL (μmol/L)	16.6 (12.8, 20.3)	15.6 (12.5, 21.6)	−0.052	0.959	
Child-pugh score (*n*)					
A	110 (95.6%)	77 (100%)	2.616	0.083	
B	5 (5.4%)	0 (0%)			
Platelet count (10^9^/dl)	132.5 (99.3,173.0)	132.5 (95.3, 179.5)	−0.371	0.711	
BCLC stage (*n*)					
0	21 (18.3%)	18 (23.4%)	0.746	0.388	
A	94 (81.7%	59 (76.6%)			
Antiviral therapy (*n*)					
Present	37 (32.2%)	33 (42.8%)	2.272	0.132	
Absent	78 (67.8%)	44 (57.2%)			
Surgical approach (*n*)					
Laparoscopic	75 (65.2%)	47 (60.1%)	0.348	0.555	
Laparotomy	40 (34.8%)	30 (38.9%)			
Type of resection (*n*)					
AR	62 (53.9%)	42 (54.5%)	0.007	0.931	
Non-AR	53 (46.1%)	35 (45.5%)			
SATI (cm^2^/m^2^)	45.7 (33.6, 58.5)	32.5 (19.7, 46.2)	−4.421	<0.001	0.749
VATI (cm^2^/m^2^)	53.5 ± 2.7	29.4 ± 13.7	7.697	<0.001	<0.001

AFP, *α*-Fetoprotein; TBIL, total bilirubin; HBV, hepatitis B virus; DM, diabetes mellitus; BMI, body mass index; AR, anatomical resection; non-AR, non-anatomical resection; VATI, visceral adipose tissue index; SATI, subcutaneous adipose tissue index; BCLC, Barcelona Clinic Liver Cancer.

**Figure 4:**
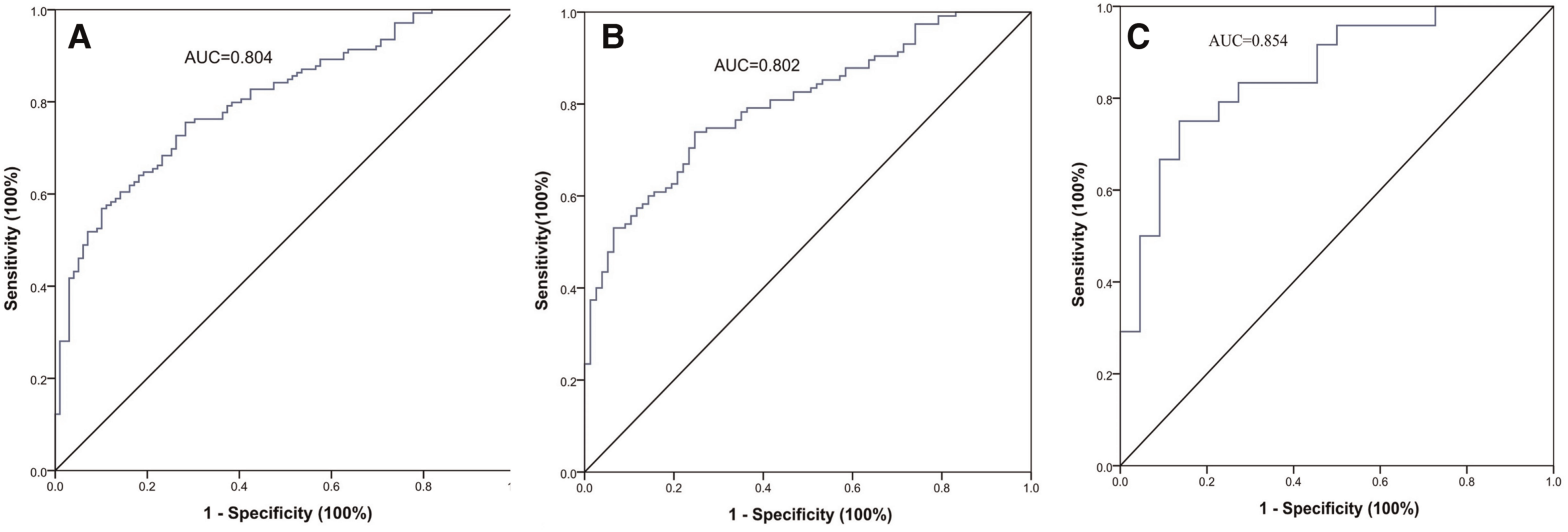
The ROC curves of the visceral adipose tissue index (VATI) for early recurrence (ER) of HBV-related HCC after hepatectomy. The AUC value was 0.804 in two cohort (**A**), 0.802 in internal training cohort (**B**), and 0.854 in external validation cohort (**C**).

**Figure 5:**
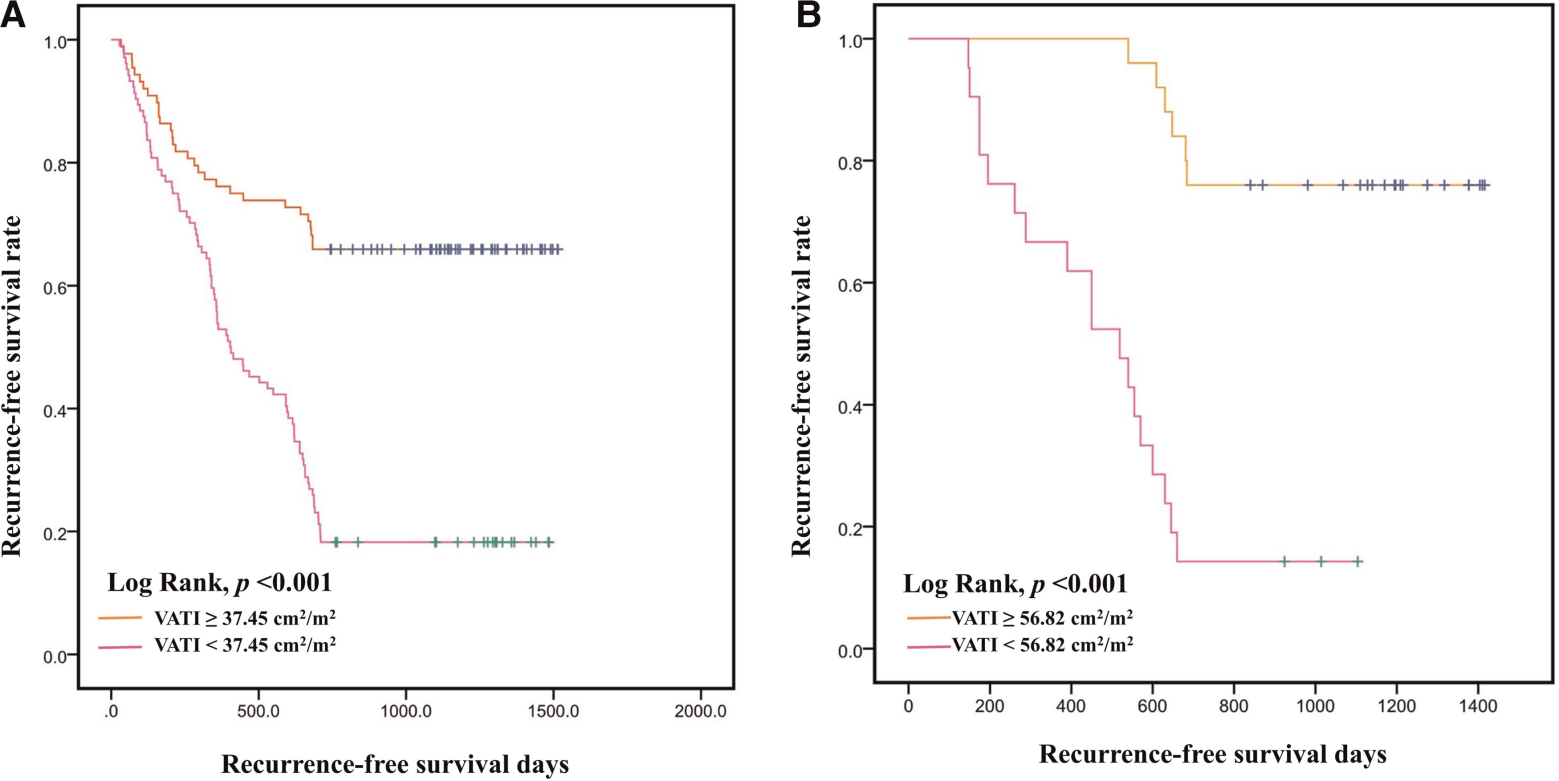
Kaplan–Meier curves for recurrence-free survival time. Recurrence-free survival with visceral adipose tissue index (VATI) ≥ 37.45 cm^2^/m^2^ and <37.45 m^2^/m^2^ in internal training cohort (**A**). Recurrence-free survival with visceral adipose tissue index (VATI) ≥ 56.82 cm^2^/m^2^ and <56.82 cm^2^/m^2^ in external validation cohort (**B**).

The AUC of the VATI was 0.802 (95% CI: 0.741–0.862, *p* < 0.001), and its sensitivity, specificity, and accuracy were 73.9%, 75.3%, and 74.6% (Figure 4), the cut-off value of VATI was 37.45 cm^2^/m^2^, then we divided into low risk (VATI < 37.45 cm^2^/m^2^) and high risk (VATI ≥ 37.45 cm^2^/m^2^) groups (Supplementary Table), and investigated the impact of VATI on PFS of HCC, the high risk group had earlier PFS compared to the low risk group (*p* < 0.001) (Figure 5).

### The effect of visceral adipose tissue index (VATI) on survival

In external validation cohort, patients were divided into ER group (*n* = 24) and non-ER group (*n* = 22). The AUC of the VATI in external validation cohort was 0.854 (95% CI: 0.745–0.963, *p* < 0.001), and its sensitivity, specificity, and accuracy were 75.0%, 86.4%, and 80.7% (Figure 4). The cut-off value of VATI was 56.82 cm^2^/m^2^, the high risk group had earlier PFS compared to the low risk group (*p* < 0.001) (Figure 5).

Furthermore, we analyzed the AUC of the VATI in two cohorts (*n* = 238), the value was 0.804, and the cut-off value of VATI was 37.45 cm^2^/m^2^, similar to internal training cohort (Figure 4).

## Discussion

Obesity is a global health issue, and the association of obesity with the development of HCC has also been reported (15, 20). Studies have shown that BMI is not an ideal measure of obesity but abdominal adipose tissue distribution, including SAT and VAT (21–23). According to our previous results, the probability of positive MVI increases with increasing VAT area (24), and this is the first study to indicate that increased VAT and SAT levels are important risk factors for ER after resection of HBV-related HCC.

The results of the present study clearly showed the first evidence that obesity-related indicators, including BMI, SATI, and VATI, are associated with ER of HBV-related HCC after curative treatment. After multivariate regression analysis, the VATI was an independent risk factor, and not BMI or SATI, which are consistent with those of a previous study (22, 25, 26). An increase in VATI value was associated with an increased risk of ER, as confirmed by the external validation group (AUC = 0.854), and the high risk group had ER compared to the low risk group (*p* < 0.001) in both cohorts. Despite this, the VATI cut-off values of the two cohorts (37.45 vs. 56.82 cm^2^/m^2^) were discrepancy, inconsistent with Imai (47.2 cm^2^/m^2^) (25) and Montano (65 cm^2^/m^2^) (22) reports as well, nevertheless, the cut-off value for VATI was similar to the internal training cohort (37.45 cm^2^/m^2^) after analyzing all patients (*n* = 238) from the two cohorts. Therefore, we speculate that the cut-off value might be closely related to the sample size of the study, and racial differences might also play a key role.

The mechanism may be VAT contains more large adipocytes secreting adipokines involved in hepatocarcinogenesis compared to SAT (23). Previous studies could speculate about the inferred explanation: (1) an increase in VAT can result in a stronger inflammatory response, which is related to the inflammatory response, and the secretion of inflammatory factors directly into the liver, eventually leading to inflammation of the liver and the development of HCC as a result (22, 27–29); (2) VAT also contributes to the development of insulin resistance, wherein insulin can exert mitogenic effects by activating its receptors in precancerous and cancer cells, which results vascular damage and vascular endothelial growth factor release, and cause oncogenesis, tumor progression and poor prognosis (15, 28, 30, 31). Therefore, as pointed out by previous studies (14, 15), pharmacological or nutritional interventions, and exercise enhancement can be used to improve chronic inflammation, reduce insulin resistance, and improve the long-term survival of patients with HCC with high VAT.

In this study, univariate analysis indicated that preoperative high AFP load was a significant risk factor for ER in HBV-related HCC, consistent with the previously reported findings (1, 10). AFP has been associated with the upregulation of proteins related to cellular infiltration and distant metastasis, promoting tumor growth and progression.

Our study has several limitations. First, this study may have overlooked other variables that could affect the results due to its retrospective design. Second, all abdominal adipose tissue were manually delineated, which may have led to some errors. Therefore, we will employ deep learning artificial intelligence methods to extract abdominal CT fat with increased accuracy in future studies. Third, this study was only conducted on HBV-related HCC. The results may not be applied to other etiologies of HCC.

In conclusion, the results of this study suggest that the VATI was an independent risk factor for ER, and higher VATI was closely related to poor outcomes after hepatectomy for HBV-related HCC (≤5 cm).

## Data Availability

The original contributions presented in the study are included in the article/**Supplementary Material**, further inquiries can be directed to the corresponding author/s.
