# Supplementary material for: Preoperative prediction of early recurrence of HBV-related hepatocellular carcinoma (≤5 cm) by visceral adipose tissue index
Source: Front Surg. 2023 Jan 6;9:985168. doi: 10.3389/fsurg.2022.985168 (PMC9852492; doi:10.3389/fsurg.2022.985168)
Supplement: Supplementary file 1 [file Table1.docx]

**Supplementary table** Baseline clinical characteristics of high risk (VATI ≥ 37.45 cm^2^/m^2^) and low risk (VATI < 37.45 cm^2^/m^2^) groups in internal training cohort

|  | **High risk** | **Low risk** |  | |
| --- | --- | --- | --- | --- |
| **Variable** | **(VATI**  **≥ 37.45 cm^2^/m^2^)**  **(n = 104)** | **(VATI**  **＜ 37.45 cm^2^/m^2^)**  **(n = 88)** | **t/z/χ^2^** | ***p*** |
| **BMI (kg/m^2^)** | 24.7 ± 2.5 | 21.5 ± 2.1 | 9.273 | < 0.001 |
| **Sex(n)**  Male  Female | 97 (93.3%)  7 (6.7%) | 68 (77.3%)  20 (22.7%) | 10.093 | 0.001 |
| **Age, years** | 51.6 (44.0, 58.0) | 49.3 (43.0, 54.0) | -1.281 | 0.200 |
| **Drinking history(n)**  Present  Absent | 34 (32.7%)  70 (67.3%) | 22 (25.0%)  66 (75.0%) | 1.365 | 0.243 |
| **Smoking history(n)**  Present  Absent | 50 (51.9%)  54 (48.1%) | 37 (42.0%)  51 (58.0%) | 0.700 | 0.403 |
| **DM (n)**  Present  Absent | 17 (16.3%)  87 (83.7%) | 5 (5.7%)  83 (94.3%) | 5.343 | 0.021 |
| **Liver cirrhosis (n)**  Present  Absent | 76 (71.0%)  28 (29.0%) | 59 (67.0%)  29 (33.0%) | 0.831 | 0.362 |
| **AFP (ng/ mL)** | 545.1  (6.4, 189.3) | 697.2  (4.6, 340.7) | -0.065 | 0.948 |
| **Albumin(g/L)** | 74.4 (70.0, 77.7) | 72.4 (69.1, 77.7) | 1.722 | 0.085 |
| **Tumor size (cm)** | 3.0 (2.2, 3.9) | 3.1 (2.0, 4.1) | -0.725 | 0.469 |
| **TBIL (μmol/L)** | 19.4 (12.6, 16.9) | 22.3 (12.8, 21.5) | -0.327 | 0.744 |
| **Child-pugh score (n)**  A  B | 100 (96.2%)  4 (3.8%) | 86 (97.7%)  2 (2.3%) | 0.390 | 0.532 |
| **Platelet count (10^9^/dl)** | 141.0  (101.3, 173.0) | 140.8  (95.3, 174.3) | 0.229 | 0.819 |
| **BCLC stage (n)**  0  A | 87 (83.7%)  17 (16.3%) | 66 (75.0%)  22 (25.0%) | 2.205 | 0.138 |
| **Antiviral therapy (n)**  Present  Absent | 37 (35.6%)  67 (64.4%) | 33 (37.5%)  55 (62.5%) | 0.076 | 0.783 |
| **Surgical approach (n)**  [Laparoscopic](C:/Users/zhangqigang/AppData/Local/youdao/dict/Application/8.9.9.0/resultui/html/index.html#/javascript:;)  Laparotomy | 71 (68.3%)  33 (31.7%) | 51 (57.9%)  37 (42.1%) | 2.189 | 0.139 |
| **Type of resection (n)**  AR  Non-AR | 56 (53.8%)  48 (46.2%) | 48 (54.5%)  40 (45.5%) | 0.009 | 0.923 |
| **SATI (cm^2^/m^2^)** | 52.2  (40.2, 60.4) | 32.2  (17.8, 42.5) | -7.327 | < 0.001 |
| **VATI (cm^2^/m^2^)** | 60.5  (17.9, 30.5) | 23.9  (43.7, 69.8) | -11.927 | < 0.001 |

AFP: α-Fetoprotein, TBIL: total bilirubin, HBV: hepatitis B virus, DM: diabetes mellitus, BMI: body mass index, AR: anatomical resection, non-AR: non-anatomical resection, VATI: visceral adipose tissue index, SATI: subcutaneous adipose tissue index, BCLC: Barcelona Clinic Liver Cancer.

**Supplementary table** Baseline clinical characteristics of high risk (VATI ≥ 37.45 cm^2^/m^2^) and low risk (VATI < 37.45 cm^2^/m^2^) groups in external validation cohort

|  | **High risk** | **Low risk** |  | |
| --- | --- | --- | --- | --- |
| **Variable** | **(VATI**  **≥ 56.82 cm^2^/m^2^)**  **(n = 21)** | **(VATI**  **＜ 56.82 cm^2^/m^2^)**  **(n = 25)** | **t/z/χ^2^** | ***p*** |
| **BMI (kg/m^2^)** | 25.0 ± 2.4 | 21.6 ± 3.0 | 9.273 | 0.000 |
| **Sex(n)**  Male  Female | 20 (95.2%)  1 (4.8%) | 22 (88.0%)  3 (12.0%) | 0.753 | 0.385 |
| **Age, years** | 61.0 ± 9.4 | 50.6 ± 12.1 | -3.176 | 0.003 |
| **Drinking history(n)**  Present  Absent | 14 (66.7%)  7 (33.3%) | 9 (36.0%)  16 (64.0%) | 3.154 | 0.076 |
| **Smoking history(n)**  Present  Absent | 15 (71.4%)  6 (28.6%) | 14 (56.0%)  11 (44.0%) | 1.166 | 0.280 |
| **DM (n)**  Present  Absent | 15 (71.4%)  6 (28.6%) | 24 (96.0%)  1 (4.0%) | 5.341 | 0.021 |
| **Liver cirrhosis (n)**  Present  Absent | 15 (71.4%)  6 (28.6%) | 18 (72.0%)  7 (28.0%) | 0.831 | 0.362 |
| **AFP (ng/ mL)** | 28.0  (5.9, 529.2) | 47.6  (4.3, 325.5) | -0.265 | 0.791 |
| **Albumin(g/L)** | 44.0 (38.5, 47.0) | 41.0 (38.0, 45.5) | -0.740 | 0.459 |
| **Tumor size (cm)** | 3.72 ± 1.23 | 2.68 ± 1.19 | -2.901 | 0.006 |
| **TBIL (μmol/L)** | 14.5 (12.0, 19.0) | 13.9 (10.5, 16.6) | -0.761 | 0.447 |
| **Child-pugh score (n)**  A  B | 21 (100.0%)  0 (0.0%) | 25 (100.0%)  0 (0.0%) | - | - |
| **Platelet count (10^9^/dl)** | 154.3 ± 58.8 | 133.2 ± 51.7 | -0.296 | 0.202 |
| **BCLC stage (n)**  0  A | 5 (28.6%)  16 (71.4%) | 16 (64.0%)  9 (36.0%) | 5.899 | 0.015 |
| **Antiviral therapy (n)**  Present  Absent | 4 (19.0%)  17 (81.0%) | 4 (16.0%)  21 (84.0%) | 0.074 | 0.786 |
| **Surgical approach (n)**  [Laparoscopic](C:/Users/zhangqigang/AppData/Local/youdao/dict/Application/8.9.9.0/resultui/html/index.html#/javascript:;)  Laparotomy | 7 (33.3%)  14 (66.7%) | 10 (40.0%)  15 (60.0%) | 0.026 | 0.873 |
| **Type of resection (n)**  AR  Non-AR | 10 (47.6%)  11 (52.4%) | 11 (44.0%)  14 (56.0%) | 0.060 | 0.806 |
| **SATI (cm^2^/m^2^)** | 46.5  (38.3, 57.9) | 28.4  (20.6, 40.6) | -3.165 | 0.002 |
| **VATI (cm^2^/m^2^)** | 81.7 ± 20.3 | 29.5 ± 14.8 | -10.048 | < 0.001 |

AFP: α-Fetoprotein, TBIL: total bilirubin, HBV: hepatitis B virus, DM: diabetes mellitus, BMI: body mass index, AR: anatomical resection, non-AR: non-anatomical resection, VATI: visceral adipose tissue index, SATI: subcutaneous adipose tissue index, BCLC: Barcelona Clinic Liver Cancer.
